# Crosstalk Between MYC and lncRNAs in Hematological Malignancies

**DOI:** 10.3389/fonc.2020.579940

**Published:** 2020-10-08

**Authors:** Kaifee Arman, Tarik Möröy

**Affiliations:** ^1^Institut de recherches cliniques de Montréal, Montreal, QC, Canada; ^2^Division of Experimental Medicine, McGill University, Montreal, QC, Canada; ^3^Département de microbiologie, infectiologie et immunologie, Université de Montréal, Montreal, QC, Canada

**Keywords:** MYC, long non-coding RNAs, leukemia, lymphoma, hematological malignancies

## Abstract

The human genome project revealed the existence of many thousands of long non-coding RNAs (lncRNAs). These transcripts that are over 200 nucleotides long were soon recognized for their importance in regulating gene expression. However, their poor conservation among species and their still controversial annotation has limited their study to some extent. Moreover, a generally lower expression of lncRNAs as compared to protein coding genes and their enigmatic biochemical mechanisms have impeded progress in the understanding of their biological roles. It is, however, known that lncRNAs engage in various kinds of interactions and can form complexes with other RNAs, with genomic DNA or proteins rendering their functional regulatory network quite complex. It has emerged from recent studies that lncRNAs exert important roles in gene expression that affect many cellular processes underlying development, cellular differentiation, but also the pathogenesis of blood cancers like leukemia and lymphoma. A number of lncRNAs have been found to be regulated by several well-known transcription factors including Myelocytomatosis viral oncogene homolog (*MYC*). The *c-MYC* gene is known to be one of the most frequently deregulated oncogenes and a driver for many human cancers. The c-*MYC* gene is very frequently activated by chromosomal translocations in hematopoietic cancers most prominently in B- or T-cell lymphoma or leukemia and much is already known about its role as a DNA binding transcriptional regulator. Although the understanding of MYC’s regulatory role controlling lncRNA expression and how *MYC* itself is controlled by lncRNA in blood cancers is still at the beginning, an intriguing picture emerges indicating that *c-MYC* may execute part of its oncogenic function through lncRNAs. Several studies have identified lncRNAs regulating c-*MYC* expression and *c-MYC* regulated lncRNAs in different blood cancers and have unveiled new mechanisms how these RNA molecules act. In this review, we give an overview of lncRNAs that have been recognized as critical in the context of activated *c-MYC* in leukemia and lymphoma, describe their mechanism of action and their effect on transcriptional reprogramming in cancer cells. Finally, we discuss possible ways how an interference with their molecular function could be exploited for new cancer therapies.

## Introduction

The knowledge gained by exploring the human genome is expanding every day thanks to high-throughput RNA sequencing (RNA-Seq) and other next generation sequencing technologies. One outcome was the discovery of thousands of non-coding transcripts (1), which were once considered transcriptional noise or “junk” (2, 3). It has now been recognized that only 2% of all transcribed regions of the human genome represent protein coding genes while the rest are non-protein coding transcripts with long non-coding RNAs (lncRNAs) being in the majority (4–7). LncRNAs are defined as transcripts longer than 200 nucleotides in length that lack open reading frames with the capacity to encode more than 100 amino acids (8, 9). Most if not all of these lncRNAs are transcribed by RNA polymerase II, are 5′ capped and polyadenylated at their 3′ ends (10). LncRNAs are classified according to their transcriptional origin and can be associated with enhancers or promoters. An additional classification is made according to the position of their genes with respect to neighboring protein-coding genes, which groups them into intronic lncRNAs, exonic/sense lncRNAs, antisense lncRNAs, intergenic lncRNAs, and bidirectional or divergent lncRNAs (11). Recent studies have shown that lncRNAs are implicated in almost all types of cancer, functioning either as tumor suppressors or oncogenes [reviewed in (12)], but in most cases a precise molecular mechanism remains yet to be discovered. Nevertheless, the relevance of lncRNAs in malignant transformation and several other important biological processes has been recognized and has placed these molecules in the center of attention (13, 14).

It is now known that lncRNAs can interact with other molecules and form RNA-RNA, RNA-DNA, or RNA-protein complexes, which renders their functional network quite complex. Although many questions remain, a number of molecular mechanisms have been unveiled that can explain how lncRNAs function. LncRNAs show interactions with chromatin modifiers such as the BRG1/BAF or the SWI/SNF complexes [reviewed in (15)] or act as modulators of protein activity or as enzyme cofactors [reviewed in (16)]. They also function as super enhancer RNAs (eRNAs) affecting multiple genes in trans (17) or by competing with miRNAs for binding to their targets (competing endogenous RNAs or ceRNAs) (18). Moreover, lncRNAs are implicated in transcriptional and post transcriptional regulation of gene expression (19) and may have direct interactions with R-loops and triple helices (20). LncRNAs often exhibit tissue- and cell-type specificity (8, 21), a feature which makes them excellent candidates for biomarkers of selected human cancers (22–25). The localization of lncRNAs within the cell closely correlates with their mode of action. LncRNAs localized in the nucleus can act in *cis* by controlling the expression of neighboring genes or in *trans* by regulating gene expression on other chromosomes or remote loci. Cytoplasmic lncRNAs have a direct regulatory function on gene expression post-transcriptionally and affect for instance mRNA stability and mRNA translation or the sequestering of proteins or miRNAs (26).

Long non-coding RNAs can directly or indirectly regulate different pathways in cancer (27), and their relevance for hematological malignancies in particular has been established at a rapid pace (28–37). Well known transcription factors and or proto-oncoproteins such as *c-* Myelocytomatosis viral oncogene homolog (MYC), *Notch1*, *beta-catenin*, or *RAS* regulate lncRNAs or are being regulated by lncRNAs in different hematological disorders (38–43), [reviewed in (29, 30, 44)]. One of the most frequently activated proto-oncogenes in human cancers is the *c-MYC* gene (*MYC* henceforth). *MYC* is a helix-loop-helix, leucine zipper (HLH-LZ) transcription factor, which dimerizes with *MAX*, a smaller HLH-LZ protein and *MYC/MAX* heterodimers bind to cognate sites containing so called E-box sequences in gene promoters and enhancers. The MYC/MAX complex has been reported to activate gene expression, albeit more recent reports also indicate that *MYC* can have a function in gene repression (45–47). *MYC* plays a significant role in many human cancers by regulating several cellular processes including cell proliferation, cell differentiation, metabolism, apoptosis, angiogenesis, and genomic stability (48–50). One of the first direct links between *MYC* and human cancers was the discovery of its role in Burkitt type B cell lymphoma. Here, a *t*(8;14) chromosomal translocation juxtaposes the coding part of the *MYC* gene to immunoglobulin μ heavy chain locus and places it under the control of the Eμ enhancer, which leads to a constitutive, transcriptional activation of *MYC* (51, 52). Besides translocations, several other mechanisms like gene amplifications and epigenetic alterations of *MYC* and posttranslational regulation of the MYC protein, in particular its phosphorylation, lead to its constitutive activation in many human cancers including hematological neoplasms (50, 53–61). *MYC* is known to target many protein-coding and lncRNA genes in different cancers (62–69), but a number studies have also pointed out that the reverse is also true and that lncRNAs regulate *MYC* activity at the post-translational level (70, 71).

It is quite pellucid that the interrelation between *MYC* and lncRNAs plays a critical role in blood cancers such as leukemia and lymphoma. Although much is known about *MYC* on one hand and lncRNA on the other in cancers, their significance of their regulatory interaction with each other in hematological malignancies is still largely unknown. In this review, we focus on the known functional interactions between *MYC* and lncRNAs in order to illustrate the intensive crosstalk that occurs between both in many hematological malignancies (Table 1). Given the importance of *MYC* for human cancers, we discuss the implications that these interactions have for new therapeutic strategies against human blood cancers.

**TABLE 1:** Summary of lncRNAs and their crosstalk with *MYC* involved in different hematological malignancies.

LncRNA	Hematological malignancy	Mechanism of action	References
**MYC-regulated lncRNAs**			
*MINCR*	BL	Controls cell cycle progression by regulating expression of MYC-targets (AURKA, AURKB) and chromatin licensing and DNA replication factor 1 (CDT1)	(36)
*DANCR*	BCL (P493 cells)	Controls cell-cycle progression by suppressing *p21* (CDKN1A)	(81)
*DANCR*	AML	Regulates key properties of cancer stem cells, such as cellular self-renewal and quiescence	(90)
*SNX29P2*	DLBCL	Unknown	(81)
*NEAT1*	CML	Modulates imatinib-induced apoptosis in CML cells	(38)
*NEAT1*	DLBC	Acts as a competing endogenous RNA (ceRNA), regulating the miR-34b-5p-GLI1 axis; promotes proliferation	(91)
*H19*	CML	Regulates *STAT5* activity that controls BCL−XL expression	(92)
*SNHG12*	NKTCL, BCL	Enhances P-glycoproteins level, lowers the sensitivity to cisplatin; promotes proliferation, possible micro RNA sponge	(73, 94)
*PVT1*	BL	Downstream effector of MYC, sustains proliferation of BL cells	(104)
**LncRNAs altering MYC activity**			
*PVT1*	ALL	Regulates *MYC* protein; promotes proliferation	(39)
*PVT1*	APL	Enhances cell proliferation by stabilizing nucleolar proteins (NOP2)*	(110)
*PVT1*, *Lilam*	AML	Stabilizes the *MYC* protein; promotes proliferation**	(111)
*PVT1*	BL	Regulates *MYC* and cell-cycle associated genes to promote proliferation	(113)
*circPVT1*	B-ALL, T-ALL	Promotes proliferation and inhibits apoptosis by regulating MYC and BCL-2 expression	(114, 123)
*SNHG12, SNHG5*	MCL	Regulates the translation initiation complex (*eIF4E*)	(125)
*KCNQ1OT1*	AML	Sponges miR-326 to regulate *MYC* expression	(131)
*UCA1*	AML	Modulates AML progression by regulating miR-296-3p/MYC axis	(140)
*CCAT1*	AML	Regulates positive feedback loop involving miR-490-3P/MAPK1/MYC	(145)
*HULC*	CML	Promotes proliferation by regulating *MYC* and *Bcl-2* protein expression and PI3K/AKT signaling pathway	(141)
*HOTAIR*	ALL	Regulates immunologic rejection of acute lymphocytic leukemia cells via Wnt/β-catenin pathway including *MYC*	(148)
*HOTAIRM1*	AML	Modulates cytarabine (Ara-C) resistance in AML via Wnt/β-catenin pathway involving *MYC*	(149)

*Mechanism involved in hepatocellular carcinoma; yet to be validated in APL. **Validated in murine model; validation in human cells still required. UN, Unknown; BL, Burkitt lymphoma; BCL, B-cell lymphoma; AML, Acute myeloid leukemia; DLBCL, Diffuse large B cell lymphoma; CML, Chronic myeloid leukemia; NKTCL, Natural Killer/T-cell lymphoma; APL, Acute promyelocytic leukemia; ALL, Acute lymphocytic leukemia; B-ALL, B-Acute lymphocytic leukemia; T-ALL, T-Acute lymphocytic leukemia; and MCL, Mantle cell lymphoma.

## MYC Regulated lncRNAs

### LncRNAs as Direct MYC Targets Are Found in Many Hematopoietic Cancers

Kluiver and his group (72) used the immortalized B cell line P493-6, which was engineered to express MYC in a tetracycline-repressible manner, and primary B-cell lymphoma samples from patients to show that lncRNAs are a main component of the *MYC*-regulated transcriptional program comparable to regular mRNAs and micro RNAs (miRNA). This study demonstrated also that both *MYC*-induced and *MYC*-repressed lncRNA genes are significantly enriched for MYC binding sites at their promoters, suggesting that lncRNA genes are indeed direct *MYC* targets. Expression profiling of lncRNAs using a custom-designed microarray allowed the identification of 1244 lncRNA loci that were candidates to be regulated by *MYC* at least in this cellular model. The authors validated *MYC* induced upregulation of 10 lncRNAs. Furthermore, the analysis of lncRNA expression in P493-6 B-cell lymphoma cells with either high or low *MYC* levels led to the identification of 498 *MYC*-regulated lncRNAs. These lncRNAs were responsive to the inactivation of *MYC* in BL cell lines, further supporting their relevance for *MYC*-driven B-cell lymphomas (72).

**FIGURE 1:**
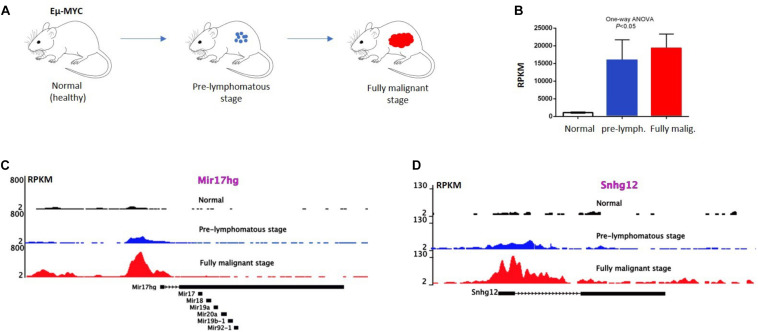
MYC occupy lncRNA gene promoters in Eμ-MYC transgenic B-cells. **(A)** Eμ MYC transgenic model, in which the tumor progression takes place. Here, a pre-lymphomatous stage exists prior to the manifestation of a fully malignant lymphoma. **(B)** Expression of MYC in the indicated B cells revealed by RNA-seq data. MYC expression in B cells positively correlates with the tumor progression at different stages of B-cell lymphoma development. Error bars, SD. *P* value < 0.05; One-way Anova analysis. **(C,D)** ChIP-seq analysis showing MYC binding to the promoter of *Snhg12* and *Mir17hg*, respectively, in normal (wild type) B-cells, in B-cells from a pre-lymphomatous animal and in B-lymphoma cells from Eμ MYC mice. Correlation of *MYC* binding to the promoters of lncRNA genes and *MYC* expression level in Eμ MYC transgenic mice (data extracted from GEO GSE51011).

Further analysis by RNA-sequencing of P493-6 B-cells (73) revealed 534 *MYC* regulated lncRNAs, 296 upregulated and 238 downregulated, in response to *MYC* overexpression. Publicly available ChIP-seq data of P493-6 cells demonstrated that *MYC*-responsive lncRNA genes were occupied by *MYC* near their transcription start sites (TSS) further supporting the notion that these lncRNAs are direct *MYC* targets. Nuclear run-on (NRO) analysis on a few selected lncRNAs also provided evidence for their direct transcriptional regulation by *MYC*. Some of these lncRNA genes (Snhg12, Snhg17, and Mir17hg) have murine homologues and the occupation of their promoters by *MYC* can be confirmed by interrogating publicly available ChIP-seq data (74) from normal, pre-lymphomatous, and fully malignant B cells from Eμ-Myc transgenic animals (Figures 1A–D). These animals carry a human *MYC* transgene, which is under the control of the strong immunoglobulin heavy chain enhancer (Eμ) and therefore express high levels of *MYC* in B cells (Figures 1A,B). The constitutive *MYC* expression drives an expansion of B cells in the bone marrow which subsequently turns to an overt, aggressive B-cell lymphoma (Figure 1A). Binding of *MYC* near the promoters of *Snhg12*, *Mir17hg* is readily detected and increases in B cells from the pre-lymphomatous and the fully malignant stage (lymphoma; Figures 1C,D). Other studies have confirmed this (73), which also suggests that the regulation of lncRNAs by *MYC* is conserved between species and is not restricted to human cells.

### The Case of MINCR

To further investigate the lncRNAs that are being regulated by *MYC* in blood cancers, several studies have chosen different strategies. The group of Ingram Iaccarino (36) analyzed RNA-seq data of 91 patients representing several B-cell lymphoma subgroups including Burkitt lymphoma, Diffuse large B-cell lymphoma (DLBCL) and Follicular lymphoma, and compared them with data from controls such as normal germinal center B cells. They also used RNA-seq data from two different *MYC*-inducible cell lines, namely hT-RPE-MycER cells, which are epithelial cells expressing a MycER fusion protein (75, 76) and P493-6 cells, with the tetracycline-repressible *MYC* allele (77, 78). This led to the discovery of 13 lncRNAs that were differentially expressed in Burkitt lymphoma carrying the *t*(8:14) Ig/MYC translocation. These lncRNAs were concordantly regulated by *MYC* in the model cell lines. Out of these 13 lncRNAs, *MINCR* (MYC-induced long non-coding RNA) showed a significant correlation with *MYC* expression in *MYC*-positive lymphomas. An siRNA mediated knockdown of *MINCR* in hT-RPE-MycER cells led to a reduction in cellular proliferation which was *MYC* independent indicating that *MINCR* acts downstream of *MYC*. RNA-seq followed by differential gene expression analysis revealed a significant enrichment of genes encoding regulators of cell cycle progression among genes that were downregulated after an RNAi mediated *MINCR* knockdown, which was in agreement with the observed defect in cell cycle progression.

While these results seemed to demonstrate that *MINCR* is a direct effector of *MYC*, several known *MYC* target genes such as Aurora kinases A and B (*AURKA*, *AURKB*), Cyclin dependent kinase 2 (*CDK2*), and Non-SMC condensing I complex subunit D2 (*NCAPD2*) were found downregulated upon *MINCR* knockdown, suggesting that *MINCR* can regulate *MYC* targets. Moreover, the promoters of 125 cell cycle associated genes that were down-regulated upon *MINCR* knockdown, showed a significant enrichment of E-boxes, i.e., sites where MYC/Max complexes contact DNA. ChIP-qPCR experiments indeed showed that *MYC* binding was reduced at all those promoter regions when the expression of *MINCR* was reduced indicating that silencing of *MINCR* might dampen MYC’s ability to engage a transcriptionally active complex at target gene promoters. However, given the complex nature of this regulatory interaction, it is presently a matter of debate whether *MINCR* is a direct *MYC*-induced lncRNA or whether *MINCR* can act back on *MYC* regulated genes; or whether both situations occur depending on cellular context (36, 79, 80). Indeed, another study has put into question whether the regulatory link between *MINCR* and *MYC* is universal, since it was not reproducible using data generated from an independent set of Burkitt lymphoma cells (79). In reply to this criticism, Doose et al. defended *MINCR* to be a *MYC*-induced lncRNA by highlighting several lines of evidence: *MINCR* is up-regulated by *MYC* in cells that contain *MYC*-inducible constructs; *MYC* occupies the promoter of *MINCR* and expression of *MINCR* and *MYC* correlates in a number of different *MYC* positive B-cell lymphomas (80). The same authors further stated that the heterogeneity typical for most cancers and cell lines, in terms of patient age, ethnicity, and effect of chemotherapy might be the reason for the lack of a general correlation between *MINCR* and *MYC* in cancer cell lines that are established in culture since long period of time (80). Along this line, the expression of *MYC* and *MINCR* did not always correlate in normal tissues and was not observed in ovarian carcinomas, chronic lymphocytic leukemia or pancreatic endocrine neoplasm (80) further supporting that the link between *MYC* and *MINCR* is context dependent and that a full clarification of the mechanistic relationship between both genes requires further experimentation.

### MYC Regulated lncRNAs as New Targets for Anti-MYC Cancer Therapies

#### DANCR and Acute Myeloid Leukemia

In a similar recent study by Lu et al. (81), 545 *MYC* regulated lncRNAs were identified via RNA-seq on P493-6 cells; many of them (238 out of 545 lncRNAs) were encoded by intergenic sequences and were found upregulated at very high levels in the malignant hematopoietic cells. Here, the lncRNA ENSG00000198106 (*SNX29P2*) was found to be specifically expressed in DLBCL. Four out of 238 MYC regulated intergenic lncRNAs were highly expressed in nearly all CCLE (Cancer Cell Line Encyclopedia) cell lines and were significantly positively correlated with *MYC* expression levels. One of which, the lncRNA ENSG00000226950, that has attracted the attention of several groups had previously been identified as a non-coding RNA associated with enhancer of zeste homolog 2 and was found to repress the runt related transcription factor 2 gene (81) and was called *DANCR* (Differentiation Antagonizing Non-Protein Coding RNA). The *DANCR* gene is bound by *MYC* when *MYC* is induced in the P493-6 cells in a time-dependent manner which is consistent with the findings of Hart et al. (73) and other studies that followed *DANCR* expression in several tumors (82–89). Of interest here is the finding that *DANCR* plays a role in Acute Myeloid Leukemia (AML) (90). AML is a heterogeneous leukemia with a hierarchical cellular organization, driven by a population of so-called leukemia stem cells (LSCs) that have an abnormally robust self-renewal capacity and increased chemotherapy resistance. RNA-seq on cytogenetically normal AML patients (CN-AML, i.e., lacking chromosomal aberrations) demonstrated that *DANCR* was specifically expressed in LSCs and the knockdown of *DANCR* resulted in the downregulation of *MYC* expression (90). These observations and the finding that disrupting *DANCR* in a murine AML mouse model prolonged the survival of the animals after serial transplantation owing to a lower self-renewal capacity and dormancy of leukemic cells made a strong case for an important functional role of this lncRNA in the maintenance or progression of AML (90). The features of *DANCR* in hematological malignancies makes this lncRNA a good candidate for a potential therapeutical application. How to target *DANCR* in AML will depend, however, on more insight into its precise molecular function in hematological disorders.

#### NEAT and H19 in CML

The lncRNA *NEAT1* (Nuclear Enriched Abundant Transcript 1) serves as a scaffold for so called paraspeckles, that are found in the nucleus at regions not occupied by chromatin. The function of paraspeckles in not well known, but their formation depends on the presence of RNA Polymerase II and is therefore linked to transcription. It was shown that *NEAT1* expression is regulated by *MYC* in DLBCL (91) and Qian and colleagues demonstrated that *NEAT1* can drive B cell proliferation and -lymphomagenesis through the miR-34b-5p-GLI1 axis and silencing of *NEAT1* dampened cell proliferation and facilitated apoptosis (91). A report from Zeng et al., indicated that *NEAT1* expression is downregulated in primary CML cells, but that its expression was rescued in K562 CML cell line when the expression or activity of the BCR-ABL kinase, that typically is present in CML cells as a consequence of the *t*(9:22) translocation, is blocked (38). In addition, the findings suggested that *NEAT1* which is regulated by *MYC* modulates imatinib-induced apoptosis in CML cells (38). A model was proposed in which pathways activated by BCR-ABL can increase *MYC* expression which leads to repression of *NEAT1* transcription. *MYC* knockdown upregulated *NEAT1* expression, and imatinib treatment of the *MYC* knockdown cells increased *NEAT1* expression further (38). Although CML can be well treated with Imatinib, resistance to this drug occurs and represents a major problem in the management of this leukemia. Of interest in this context is the observation that knockdown of *NEAT1* renders K562 CML cells more sensitive to Imatinib and proteasome inhibitors, since combinatorial treatment leads to accelerated apoptosis of K562 cells. Understanding the link between *BCR-ABL*, *NEAT1*, *MYC*, and the sensitivity of CML cells to Imatinib will provide further insight into the mechanisms of Imatinib resistance and ultimately lead to a way to circumvent it.

Similar to *NEAT1, H19*, a maternal lncRNA, is expressed in CML cells with the *t*(9:22) translocation and its expression depends on the presence of the BCR-ABL fusion protein, since a knockdown of BCR−ABL expression led to a downregulation of *H19* expression in K562 cells (92). Treatment with imatinib also decreased the level of *H19* in K562 cells, further confirming that the expression of *H19* is BCR−ABL kinase dependent. Again, as for *NEAT1*, knockdown of *H19* significantly increased the apoptosis in K562 cells following imatinib treatment pointing to the possibility of a way to enhance the drug’s effectiveness. Further studies into the underlying mechanisms demonstrated that *H19* may have an impact on leukemic cell survival by regulating STAT5 activity, an anti-apoptotic protein that stimulates expression of pro-survival factors such as BCL-XL. *In vivo* experiments involving subcutaneous injection of K562 cells engineered with a stable knockdown of *H19* into mice demonstrated the tumorigenic role of *H19*, since a significant decrease of tumor growth was observed (92). Moreover, the expression of *H19* was found to be *MYC* dependent in K562 leukemic cells. Treatment with imatinib resulted in a decreased level of *MYC* mRNA similar to *H19*. A knockdown of *MYC* downregulated *H19* expression while the ectopic over-expression of *MYC* upregulated *H19* levels in K562 cells, regardless whether they were treated with imatinib or not, supporting the notion that the expression of *H19* is *MYC*-dependent (92). It is therefore thought that the disruption of *H19* expression along with imatinib treatment would be a promising therapeutic strategy to combat CML, as the combination of these two could trigger leukemic cells to undergo apoptosis. However, as for the action of *NEAT1* further studies on *H19* are required to better understand this combination strategy in order to have continuous positive effect with minimal imatinib resistance in CML.

#### SNHG12 in Natural Killer/T-Cell Lymphoma

**FIGURE 2:**
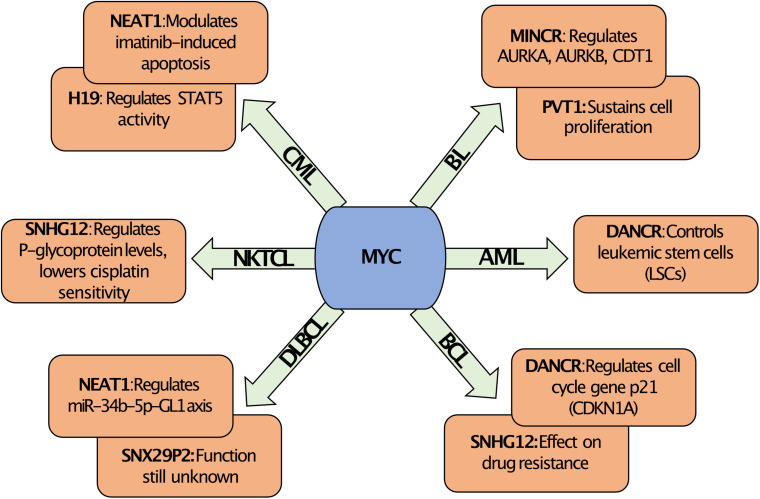
MYC regulated lncRNAs. Overview of *MYC* regulated lncRNAs along with their known biological function in different hematological malignancies. The regulation of the expression of specific lncRNAs by *MYC* is context dependent and is specific for the hematological malignancy. For example, *MYC* regulates *DANCR* in AML, which affects leukemic stem cells, but in BCL the regulation of *DANCR* by *MYC* targets the function of the negative cell cycle regulator p21 (*CDKN1A*). Similarly, *MYC* can regulate the expression of *NEAT1* in CML or DLBCL, but this affects Imatinib response or the miR-34b-5p-GL1 axis, respectively. In contrast, *MYC* can regulate *SNHG12* in BCL or in NKTCL, which in both cases can alter drug resistance. Abbreviations: BL, Burkitt lymphoma; BCL, B-cell lymphoma; AML, Acute myeloid leukemia; DLBCL, Diffuse large B cell lymphoma; CML, Chronic myeloid leukemia; and NKTCL, Natural Killer/T-cell lymphoma.

The lncRNA *SNHG12* (small nucleolar host gene 12), also known as LINC00100/ASLNC04080 is located on chromosome 1 in humans and is suspected to play a pivotal role in a number of cancers including blood cancer [reviewed in (93)]. It was noticed that the expression of *SNHG12* was higher in Natural Killer/T-cell lymphoma (NKTCL) tissues as compared to controls, in this case reactive hyperplasia of lymph node (RHLN) tissues. Moreover, *SNHG12* showed more expression in NKTCL tissues of advanced stages (3 and 4) compared to earlier stages 1 and 2, indicating its positive correlation with clinical grading (stage in 3/4) of NKTCL. *MYC* was shown to act directly on the *SNHG12* gene and the expression of *MYC* positively correlated with the expression of *SNHG12* in NKTCL (94). Both *MYC* and *SNHG12* acted in a similar fashion in NKTCL by promoting proliferation, enhancing the level of P-glycoproteins (P-gp) that are linked to multi drug resistance (MDR) proteins and inhibiting the sensitivity to cisplatin (CCDP) (94). Besides, *MYC* was found to bind directly to the promoter of *SNHG12* and to upregulate its expression. Rescue experiments further demonstrated that the overexpression of *SNHG12* was able to partially rescue the negative effects of *MYC* knockdown on drug resistance and on cell proliferation (94). The results found by Zhu et al. indicated that the regulation of *SNHG12* by *MYC* might be an important factor for the chemotherapy resistance for NKTCL (94). Therefore, loss of function of *SNHG12* could possibly overcome or defer tolerance of NKTCL to therapy, which would establish the lncRNA *SNHG12* as a therapeutic target for controlling MDR in NKTCL. RNA-seq data from a study using with P493-6 revealed that *SNHG12* can also be upregulated by *MYC* in human B cells confirming the findings in NKTCL (73). A positive correlation between *MYC* and *Snhg12* was also seen in the Eμ-*MYC* transgenic lymphoma model (see Figure 1) adding support to the notion that *Snhg12* might have important roles in other hematopoietic malignancies besides NKTCL. An overview of all the above described *MYC*-regulated lncRNAs in different hematopoietic malignancies is shown in Figure 2.

## LncRNAs That Alter MYC Activity

Since a constitutive activation or overexpression of *MYC* plays an exceptionally important role in many, if not all, hematological malignancies, a tight regulation of this proto-oncoprotein in normal cells is essential to avoid a derailment of physiological processes such as cell cycle progression, cell death, or metabolic pathways (49, 95). To achieve this, the expression and the activity of *MYC* is controlled by multiple mechanisms beyond the control of *MYC* gene expression at the transcriptional level. The multitude of regulatory mechanisms include post-translational modifications that alter MYC’s stability and half-life and the interaction with a large number of partner proteins that modify MYC’s activity and also its capacity to bind DNA (96, 97). Genes for several non-coding RNAs including both miRNA and lncRNAs are located in the vicinity of the *MYC* gene locus where they play important roles in the regulation of *MYC* (98–100) in both normal cells and in hematological malignancies. Here, we discuss in more detail those lncRNAs that regulate *MYC* activity in hematological malignancies. One of the best studied lncRNAs that exerts such activities is *PVT1*.

### LncRNAs Regulating MYC as New Targets for Blood Cancer Therapies

#### The Link Between MYC and PVT1

**FIGURE 3:**
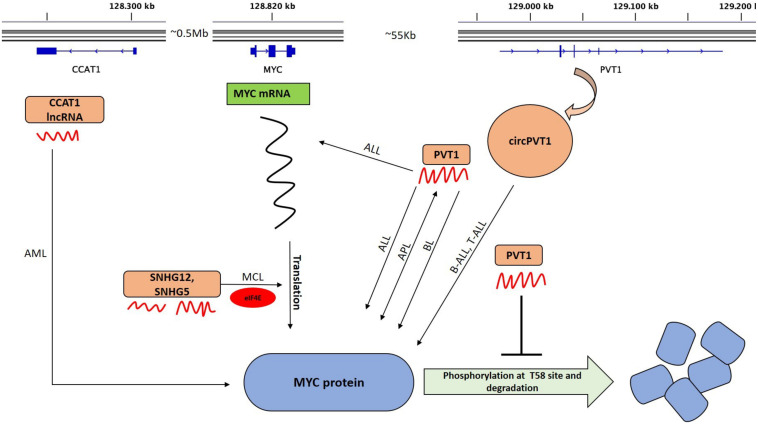
LncRNAs that alter MYC activity. The upper part shows the graphical representation of the genomic location of *MYC*, *PVT1*, and *CCAT1*. The transcribed lncRNAs (curved red structures) regulate *MYC* at mRNA or protein level and some at both. Those that regulate *MYC* mRNA translation in different hematological malignancies are indicated. Positive feedback interaction between lncRNA and *MYC* is also shown (double headed arrow). *circPVT1* is transcribed from exon 2 (E2) of *PVT1*. *PVT1* maintains a high MYC protein level and prevents the degradation of *MYC* by blocking the phosphorylation of Thr58 in *MYC*. *SNHG5* and *SNHG12* modulate *MYC* translation in MCL cells via translation machinery through eIF4E. LncRNA *CCAT1* interacts with *MYC* in AML. BL, Burkitt lymphoma; AML, Acute myeloid leukemia; APL, Acute promyelocytic leukemia; ALL, Acute lymphocytic leukemia; B-ALL, Acute lymphocytic leukemia; and MCL, Mantle cell lymphoma.

Plasmacytoma Variant Translocation 1 (*PVT1*) is a lncRNA that is known since a long time and was linked to *MYC* shortly after its discovery. The gene encoding *PVT1* is located on the long arm of chromosome 8q24 in a region about 55 kb distal to the *MYC* gene (Figure 3) and, very similar to *MYC*, is frequently involved in translocations occurring in Burkitt lymphoma (101, 102). *PVT1* functions in close coordination with *MYC* and the intricate relationship between both genes was recently explained in a new study (70), which showed a *PVT1* dependence for those cancers that have a copy-number increase of *MYC*. It is known that *MYC* is consistently co-gained with the adjacent genes *PVT1, CCDC26*, and *GSDMC* that lie within a region of about 2 Mb (70). To explore whether a low copy-number gain of these genes promotes tumorigenesis or not, three strains of transgenic mice were created; one with single copy of *MYC* alone, another one with the region containing *PVT1*, *CDC26*, and *GSDMC* and third line that carried the entire 2 Mb syntenic region including *MYC*, *PVT1*, *CDC26*, and *GSDMC*. All three lines showed normal phenotypes with no developmental abnormalities. However, a single copy of *MYC* was insufficient to accelerate *MMTV-Neu* driven tumorigenesis and a combination of both *MYC* and *PVT1* was required (70) indicating a collaborative relationship between both genes (Figure 3).

Analysis of data from The Cancer Genome Atlas (TCGA) database revealed that a co-gain of both *MYC* and *PVT1* in almost all (98%) tumors showing 8q24 copy-number increases, whereas very few (0.15%) tumors showed an increase in copy number of *MYC* alone without *PVT1*. CRISPR Cas9-mediated disruption of *PVT1* in *MYC* dependent HCT116 colon cancer cells significantly reduced its tumorigenic potency suggesting that *PVT1* regulates the activity of *MYC* as an oncogene (70) or even act as an oncogene independently of *MYC* (103). Several studies provided data supporting a direct regulation of *MYC* by *PVT1*, which is discussed further below. However, Carramusa et al. (104) proposed that, in contrast, *MYC* transcriptionally activates *PVT1* and *PVT1* is a downstream effector of *MYC* in Burkitt lymphoma. Their finding that the *PVT1* gene locus bears specific E-boxes allowing binding of *MYC* to bind to the *PVT1* promoter region supported this view (104).

##### The MYC – PVT1 feedback loop mechanism

Since *PVT1* also acts as a *cis* regulatory lncRNA (70, 105–108), the interaction between *MYC* and *PVT1* in different hematological malignancies has to take place via a positive feedback loop (109, 110). To exert its oncogenic activity via *MYC*, *PVT1* prevents the degradation of *MYC* by hindering its phosphorylation at threonine 58 resulting in increased *MYC* stability (Figure 3), possibly by a direct binding or interaction with this amino acid residue (70, 107). This causes a feed forward loop since the stabilization of *MYC* enhances the expression of *MYC* target genes among them also *PVT1*, which leads to even more active *MYC*. Support for this comes from studies with acute promyelocytic leukemia (APL) cell lines, where knockdown of *PVT1* resulted in the downregulation of *MYC* whereas *MYC* silencing also reduced *PVT1* levels, causing reduced proliferation (110). *PVT1* also regulates *MYC* in acute lymphoblastic leukemia (ALL) where *PVT1* can act as an oncogene and drives both the development and progression of this type of blood cancer (39). Experiments in Jurkat cells (a human acute T cell lymphoblastic leukemia cell line) showed downregulation of MYC both at mRNA and protein levels upon siRNA mediated knockdown of *PVT1* resulting in accelerated apoptosis, slower proliferation rates and a G0/G1 cell cycle arrest. These effects were attributed to the degradation of MYC and BCL2, which increased levels of caspase-3 and the negative cell cycle regulators and tumor suppressor genes CDKN2A and -2B (p16INK4a and p15INK4b) (39). All these data suggest that *PVT1* is required for the development of ALL (Figure 3) and successful interference with *PVT1* could offer new possibilities for a targeted therapy against ALL (39). Since *MYC* depends on *PVT1* in its ability to drive malignant transformation and is itself difficult to target because it lacks enzymatic activity, several groups have speculated about the validity and feasibility to target *PVT1* to weaken or inhibit *MYC* in tumor cells.

In murine MLL-ENL AML models, depletion of *Pvt1* together with two other lncRNAs, called *Lilam* (leukemia-induced lncRNA affecting *Myc*) and *Pilna* (progenitor-induced lncRNA neighboring Ak3), activated a myeloid differentiation program which was reversed by *Myc* overexpression showing an epistatic relationship between these three lncRNAs and *Myc* (111). In addition, the *PVT1* promoter also has tumor suppressor activity independently of the *PVT1* lncRNA. In fact, both *PVT1* and *MYC* promoters compete for four intragenic enhancers in the *PVT1* locus. Here, the *PVT1* promoter functions as a DNA boundary element, inhibiting *MYC* from accessing its downstream enhancers and finally hindering the transcription of *MYC* and impeding its oncogenic functions (112). In another study, knockdown of *PVT1* inhibited the proliferation of Burkitt lymphoma cells by arresting the cells in G0/G1 phase which was associated with a reduction of *MYC* expression and alterations in the expression of cell cycle-associated genes. A cell cycle PCR array showed that 54 genes including *CCNG2* (Cyclin G2), *RBL2* (Retinoblastoma-like 2), *CDKN1A* (Cyclin-dependent kinase inhibitor 1A) and others were upregulated while 26 genes such as *CCNE1* (Cyclin E1), *CCND1* (Cyclin D1) and *CDC20* (Cell division cycle 20) were downregulated. The authors concluded that the silencing of *PVT1* retarded the proliferation of Raji cells though a downregulation of *MYC* expression and subsequent alterations in the expression levels of cell cycle-associated genes (113).

Another intriguing property of the *PVT1* gene locus is the fact that it also encodes a circular RNA called *circPVT1* (Figure 3) with 26 different isoforms (114). The most common isoform which has the highest level of expression in blood cells includes the whole exon 2 of *PVT*1, which forms a closed loop-like structure (114–116). Circular RNAs (circRNAs) have been identified as a new class of non-coding RNAs which are well conserved, widespread, abundant and are regulated independently of their cognate linear isoform in the eukaryotic transcriptome (115, 117, 118). These are generally formed by back-splicing of pre-mRNA and form a closed loop structure in which their 3′ and 5′ ends are covalently linked conferring them an increased stability and resistance (115, 119, 120). LncRNA *PVT1* and *circPVT1* regulate their expression independently of each other as they arise from separate individual promoters and have different genome localizations (114, 121, 122). The role of *circPVT1* and *PVT1* in different hematological malignancies with downstream deregulation of *MYC* has been very well highlighted in a recent review by Ghetti et al. (114). *circPVT1* is well studied in ALL in which the authors demonstrate that *circPVT1* (but not *PVT1*) was specifically highly expressed in human patient samples of ALL but not AML samples as compared to healthy control groups (123). Knockdown of *circPVT1* had no effect on its mother gene *PVT1* but significantly decreased the protein levels of neighboring genes, MYC and *BCL2* suggesting that *circPVT1* might enhance *BCL2* expression to inhibit *MYC* mediated apoptosis (123). The authors speculate that *circPVT1* might be acting as competing endogenous RNAs (ceRNAs) of miR-let-7 and miR-125 as the expression of these miRNAs were reduced in the ALL patients through the sponging effect of *circPVT1* (123). These two miRNAs also target *MYC* and *BCL2* and their lower expression reduces their inhibitory effect on *MYC* and *BCL2* resulting in expansion of ALL (123). The above cited examples indicate that the specific functions of *PVT1* and *circPVT1* in hematological malignancies are with some certainty caused by the downstream deregulation of *MYC*. Interference with *PVT1* RNAs would therefore have a direct effect on *MYC* expression, which makes them potential therapeutic targets.

Colon cancer associated transcript1 (*CCAT1*) is a another lncRNA present along in the same genomic region as *PVT1* and *MYC* (Figure 3). *CCAT1* is implicated in multiple solid tumors and is generally viewed as a dominant oncogene. High *CCAT1* expression seems to induce a block of myeloid differentiation; a situation which can be seen as a prerogative of myeloid transformation and the emergence of an AML. Furthermore, both *CCAT1* and *PVT1* expression was found to be over 5 fold higher in *t*(8;21) positive versus *t*(8;21) negative AML samples and was associated with clinical parameter indicating higher risk and lower overall survival of AML patients positive for this translocation (124). Similar to *PVT1*, the promoter of *CCAT1* is occupied by *MYC* and *MYC* can upregulate *CCAT1* expression. It therefore can be inferred that part of MYC’s oncogenic potential is mediated indirectly through the action of *CCAT1* and *PVT1*. These findings support a role of *CCAT1* and *PVT1* as dominant oncogenes in AML and suggest that interference with the function of these lncRNAs could represent a new therapeutic tool against *MYC* driven hematological malignancies (124).

#### LncRNA Acting on the Translation of MYC mRNA

The translation of mRNAs and, in particular, ribosomal entry is regulated in part by a group of proteins called “eukaryotic translation initiation factors” or eIFs. In lymphoma cells, lncRNAs exist that can regulate the translation of the *MYC* specific mRNA in cooperation with the eukaryotic translation initiation factor 4E (*eIF4E*). Recently, Mamta and her group identified several other translation-machinery associated lncRNAs in mantle cell lymphoma (MCL) (125). To find lncRNAs associated with the translation initiation complex, RNA Immunoprecipitation and sequencing (RIP-seq) followed by RNA-IP using an anti-eIF4E (translation initiation factor-4E) antibody was performed using samples from MCL patients, normal controls and MCL cell lines (125). The lncRNA *SNHG12* which was also shown to be regulated by *MYC* (see above) and lncRNA *SNHG5* turned out to be highly enriched in the precipitates. Knockdown of *SNHG12 or SNHG5* significantly increased the protein levels of MYC indicating that these lncRNAs can modulate *MYC* expression level by altering its translation in MCL (125). It is therefore be concluded that among the bona fide lncRNAs that interact with the translation machinery through *eIF4E*, i.e., both *SNHG5* and *SNHG12*, can modulate *MYC* translation in MCL cells (125) and thus strongly suggesting that these lncRNAs regulate the translation of *MYC* (Figure 3).

#### LncRNAs and MYC: The Micro RNA Connection

**FIGURE 4:**
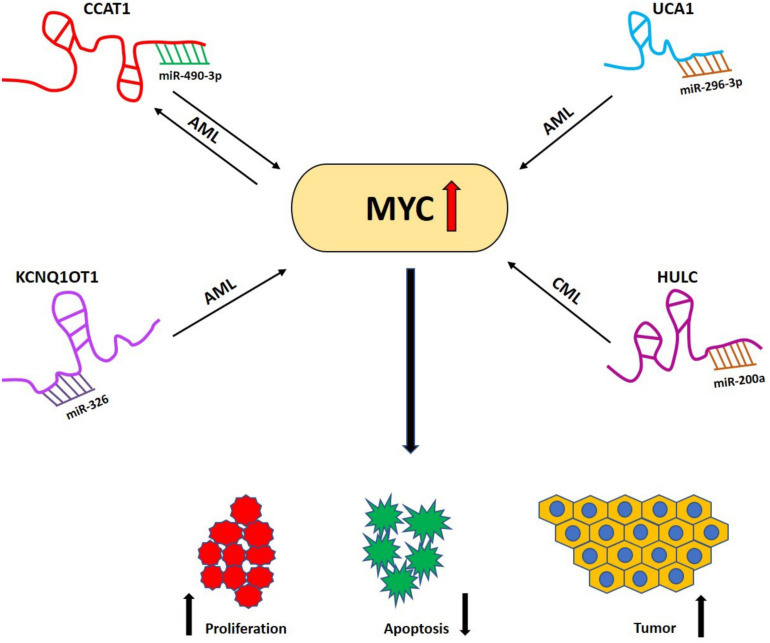
LncRNA/miRNA/MYC axis in different hematological malignancies. Different lncRNAs acting as competing endogenous RNAs (ceRNAs) sequestering miRNAs, which results in the elevation of *MYC* expression and eventually enhances its tumorigenic effect (decreased apoptosis, increased proliferation and tumor progression) in different hematological malignancies. LncRNA *CCAT1* acts as a sponge for miR-490-3p and generates a feedback loop mechanism in AML. Other lncRNAs such as *UCA1*, *KCNQ1OT1* sequester miR-296-3p and miR-326, respectively, in AML while lncRNA *HULC* sponges miR-200a in CML. AML, Acute myeloid leukemia; CML, Chronic myeloid leukemia.

Among the most interesting observations made by studying non-protein coding RNAs was that lncRNAs and miRNAs can compete for shared binding sequences and that this competition affects the expression of specific target genes. In this case, lncRNAs that function as competitive endogenous RNAs are designated “ceRNAs”; they sequester miRNAs which directly affects the miRNA target gene. The ceRNA networks involving lncRNAs, miRNAs and protein coding mRNAs affect a wide spectrum of biological processes. Relevant for this review is the lncRNA/miRNA/MYC axis, because it is involved in hematological malignancies (Figure 4). An example is the ubiquitously expressed lncRNA *KCNQ1OT1*, which is transcribed in an antisense direction from intron 11 of the *KCNQ1* gene and is localized in the nucleus (126). *KCNQ1OT1* is involved in epigenetic gene silencing (127), controls maternal *CDKN1C (p57^Kip2^*) expression in muscle cells by promoting accumulation of *H3K27me3* at its promoter, which is catalyzed by the histone-methyl-transferase *EZH2* (128). Knockdown of *KCNQ1OT1* led to the upregulation of the maternal and functional *p57*^Kip2^ allele during muscle differentiation (128). Previous evidence had already indicated that this lncRNA can act as an oncogene in AML via different mechanisms (129, 130). In one recent study, *KCNQ1OT1* was shown to regulate *MYC* expression by sponging miR-326 in AML (131). Knockdown of *KCNQ1OT1* significantly decreased MYC protein expression in AML cell lines which was rescued by miR-326 abrogation. These findings indicated that *KCNQ1OT1* is a so called “competing endogenous” RNA (ceRNA) for miR-326 to regulate *MYC* expression in AML cells. Other loss of function studies showed that *KCNQ1OT1* is required for cell proliferation and survival and that loss of *KCNQ1OT1 promotes* cell differentiation in AML cells, a feature which makes it attractive as a therapeutic target for this type of leukemia (131).

The lncRNA urothelial carcinoma associated 1 (*UCA1*) was discovered by Wang et al. as a specific and possibly unique biomarker for bladder carcinoma (132). However, it was also found to be overexpressed in different other malignancies (133–135) and mounting evidence shows its carcinogenic role in AML (136–139). Li et al. discovered the existence of a miR-296-3p/MYC axis in AML being regulated by *UCA1* (140). Here, *UCA1* acted as a ceRNA of miR-296-3p by binding to miR-296-3p. *MYC* is a target of miR-296-3p and *UCA1* positively regulates *MYC* expression. The study also carried out *in vivo* experiments in which HL-60 cells with stable *UCA1* knockdown were injected subcutaneously into the armpit of NOD/SCID mice (140). It was found that *UCA1* knockdown led to significant reduction in tumor volume. The expression analysis in the tumors showed miR-296-3p to be significantly upregulated while *MYC* was downregulated both at mRNA and protein levels that complements their findings with *in vitro* assay (140).

Work from the Wang group established the lncRNA *HULC* as a novel candidate that regulates *MYC* in CML (141). *HULC* is well conserved in primates; its gene is located on chromosome 6p24.3. The *HULC* lncRNA is spliced, polyadenylated and is located in the cytoplasm (142, 143). In bone marrow samples from CML patients, the expression levels of *HULC* correlated positively with *MYC* and knockdown of *HULC* retarded the proliferation of CML leukemic cells and induced apoptosis by repressing *MYC*, *Bcl-2* and by upregulating the *PI3K/AKT* signaling pathway (141). Moreover, *HULC* can function as a ceRNA for miR-200a to modulate *MYC* and *Bcl-2* expression in CML cells; however, whether *HULC* is a new target for CML therapy needs still to be validated in particular using animal studies.

##### CCAT1/miR-490-3p/MAPK1/MYC axis involving a feedback loop mechanism in AML

Colon cancer−associated transcript−1 has been studied and shown to play important roles in AML (124, 144). Recently Wang et al. revealed the existence of a feedback loop mechanism involving CCAT1/miR-490-3p/MAPK1/MYC in AML (145). They demonstrated that *CCAT1* acted as an oncogene promoting the proliferation and inhibiting the apoptosis while miR-490-3p acted as tumor-suppressor with opposite phenotypes in AML cells. *CCAT1* acted as sponge for miR-490-3p to elevate *MAPK1* and *MYC* expression (Figure 4). Overexpression of *MYC* upregulated the expression of *CCAT1* and *p-MAPK1* while downregulated the expression of miR-490-3p. Upon silencing *CCAT1*, expression of *p-MAPK1* and *MYC* was significantly decreased indicating that a positive feedback loop exists among CCAT1/miR-490-3P/MAPK1/MYC in AML cells and therefore provided new candidates for future research and treatment of AML (145).

#### LncRNAs in HOX Gene Clusters: HOTAIR and HOTAIRM1

**FIGURE 5:**
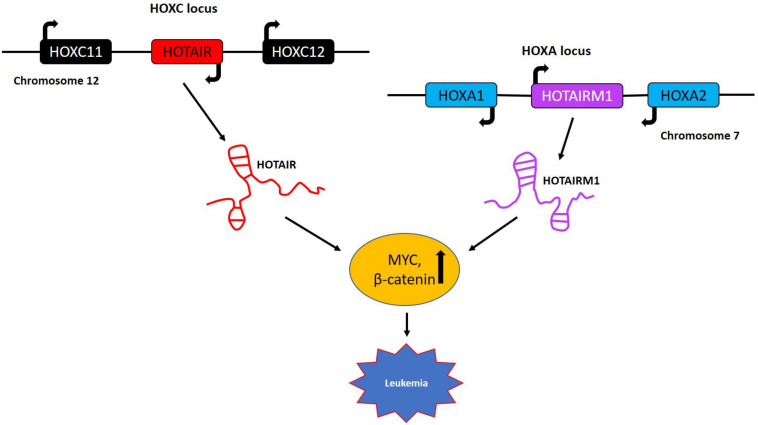
LncRNAs in HOX gene clusters with role in leukemia. *HOTAIR* is generated by transcription of the antisense strand of the *HOXC* gene, which is specifically located between *HOXC11* and *HOXC12* on chromosome 12. The lncRNA *HOTAIRM1* is transcribed from the antisense strand of the *HOXA* gene cluster and is located between the *HOXA1* and *HOXA2* genes on chromosome 7. These two lncRNAs primarily regulate the expression of *MYC* and β*-catenin* and are implicated in lymphocytic leukemia and acute myeloid leukemia, respectively.

HOX genes are found in clusters on different chromosomes and encode several important lncRNAs most notably, *HOTAIR*, *HOXA11-AS*, *HOTTIP*, and *HOTAIR* myeloid 1 (*HOTAIRM1)* (146). Among these, *HOTAIR* is one of the most well-studied lncRNAs arising from the *HOXC* gene cluster on chromosome 12 (Figure 5). This lncRNA is upregulated in the majority of cancers and acts as an oncogene [reviewed in (147)]. To study the role of *Hotair* in the immunologic rejection of leukemic cells, DBA/2 mice were injected intravenously with cells from the L1210 lymphocytic leukemia cell line (148). Leukemic mice were then treated with *Hotair* mimics as well as small interfering RNA against *Hotair*. Interestingly, the Wnt/β-catenin pathway was activated and the expression of *Myc*, *cyclinD1*, and *GSK−3*β were increased in the bone marrow of these mice induced by overexpressing *Hotair* while knockdown of *Hotair* had the opposite effects. One of the major conclusions of this study was that *Hotair* inhibits the immunologic rejection of acute lymphocytic leukemia cells in mice by activating the Wnt/β-catenin pathway including *Myc* as one of its effectors (148).

*HOTAIR* myeloid 1 is another lncRNA belonging to the *HOXA* gene cluster with a specific role in the maturation of myeloid cells and possibly in AML (Figure 5). Most recently, a potential role of *HOTAIRM1* as a therapeutic target for overcoming the cytarabine (Ara-C) resistance in AML was reported (149). These studies showed that knockdown of *HOTAIRM1* in the HL60 and THP-1 leukemia lines inactivated the Wnt/β-catenin pathway by downregulating the expression of β-catenin, *PFKP* (platelet-type phosphofructokinase) and *MYC*. In addition, *HOTAIRM1* knockdown enhanced cytarabine cytotoxicity via the Wnt/β-catenin pathway also involving *MYC* in HL60 cells (149). The *HOX* gene clusters harbor several lncRNAs regulating *MYC* expression and even protein coding genes from *HOXA* and *HOXC* gene clusters like *HOXA9*, *HOXA10*, and *HOXC6* have been shown to play crucial roles in AML and ALL by regulating *MYC* (150, 151). The above examples indicate the involvement of a wide spectrum of genes from the HOX gene clusters in the regulation of MYC in different hematological malignancies offering a series of alternative targets for future therapies.

## Conclusion and Future Perspectives

The relationship between the proto-oncoprotein MYC, the *MYC* gene and lncRNAs is of high complexity since they form an interactive network in which all molecules involved can be regulators or targets connected by linear dependencies and feed-back or feed-forward loops. This network between *MYC* and lncRNAs clearly plays a crucial role in the regulation of gene expression in different hematological malignancies and a number of ongoing studies will reveal additional lncRNAs that regulate or are controlled by *MYC*. Since *MYC* is activated in a majority of human cancers and in many malignancies of the hematopoietic system, targeting this transcription factor would be a powerful approach for an effective tumor therapy. However, despite many efforts direct targeting of *MYC* remains challenging because of its “undruggable” protein structure, lack of enzymatic activity and its nuclear localization, rendering it less accessible as for example membrane receptors or cytoplasmic kinases. Therefore, alternative possibilities have to be explored such as targeting pathways that are involved in the regulation of *MYC* expression, or the activity or stability of the MYC protein. First studies have now appeared describing how to achieve effective targeting of lncRNAs and several different methods have emerged. One possibility remains the direct transcriptional inhibition of lncRNAs through Crispr/Cas9 mediated disruption or silencing via siRNAs. However, because lncRNAs interact with proteins, an alternative strategy would be to interrupt this interaction with small molecules, which could then be developed into drug candidates (152). The future will show which method will prevail, but given the importance and critical role of lncRNAs that regulate *MYC* or act as *MYC* effectors, their role as therapeutic targets cannot be underestimated. It is well possible that they represent a new way to control *MYC* activity indirectly and lead to successful strategies to neutralize MYC and offer promising as well as intriguing new therapeutic approaches where previous concepts have not been successful.

## Author Contributions

KA and TM have generated the text and the figures. Both authors contributed to the article and approved the submitted version.

## Conflict of Interest

The authors declare that the research was conducted in the absence of any commercial or financial relationships that could be construed as a potential conflict of interest.
